# Intersecting challenges: Substance use and mental health disparities across self-reported disability status

**DOI:** 10.1016/j.josat.2025.209857

**Published:** 2025-12-08

**Authors:** Jessica Williams, Xiao Li, Hannah S. Szlyk, Erin Kasson, Nathaniel A. Dell, Alex Ramsey, Patricia A. Cavazos-Rehg

**Affiliations:** a Department of Psychiatry, Washington University School of Medicine, 660 South Euclid Avenue, Box 8134, St. Louis, MO 63110, United States

**Keywords:** People with disabilities, Disability status, Barriers to care, Substance use disorder, Substance use, Substance use treatment, Accessibility in mental health treatment

## Abstract

**Purpose:**

Substance use and substance use disorders (SUD) disproportionately affect people with disabilities (PWD), yet PWD remain under-represented in addiction treatment literature. While recent work has begun to address SUD prevalence and recovery service gaps, little is known about how disability status shapes substance use outcomes. The present study is the first to our knowledge to investigate how recovery, mental health comorbidities, and barriers to care differ across disability status using structured survey data from people who use substances.

**Methods:**

333 participants who use substances and provided information about their disability status in the baseline survey for a digital intervention study were included in the analysis (Mean_Age_: 41.1 (10.6); 58.0 % female; 68.2 % White). Bivariate analyses—independent sample *t*-tests and Chi-Square tests—were conducted to examine differences across disability status in recent substance use patterns, mental health comorbidities, recovery history, and barriers to treatment. Multivariable regression models were used to assess associations between disability status and variables of interest, adjusting for socio-demographic covariates.

**Results:**

Of the participants, 34.2 % reported having a disability (*n* = 114). PWD were more likely to be of color, unemployed, insured, and older, compared to those without disabilities (*n* = 219; ps ≤0.001). Regression analyses indicated that PWD had significantly higher odds of using cannabis, alcohol, stimulants, and other drugs over past 30-day (aORs ranged 2.22–2.30). PWD also had higher odds of endorsing depression, anxiety, and lifetime suicide attempts (aORs ranged from 2.39 (anxiety) to 3.38 (depression)). Additionally, PWD perceived more barriers to substance use treatment (β: 0.55 (0.24)) and reported more overdoses (aOR (95 % CI): 2.60 (1.49, 4.54)) and prior recovery attempts (aOR: 2.69 (1.55, 4.68)), compared to those without disabilities.

**Conclusions:**

This study is among the first to use quantitative survey data to assess and compare substance use and treatment engagement among PWD and their non-disabled peers. Findings underscore urgent equity gaps in SUD treatment systems and the importance of incorporating disability-informed frameworks in clinical assessments and recovery services. These insights complement recent qualitative findings and emphasize the need for inclusive, accessible, and person-centered care approaches in substance use health services and research.

## 1. Introduction

Substance use and substance use disorders (SUD) are recognized as major public health challenges contributing to the nation’s overall health burden (Spencer, Garnett, & Miniño, 2024). Further, accidental overdoses are the leading cause of unintentional deaths in the U.S. (Ahmad, 2024). As such, substance use is a significant public health crisis with vast consequences. Recent findings indicated that 59.0 % of Americans aged 12 and older had used some substance in the last month (including tobacco/nicotine, marijuana, alcohol, and illicit drugs) and 17.1 % met criteria for an SUD (Daniel et al., 2024). The ubiquitous nature of this crisis suggests the need for inclusive and equitable substance use research—especially among underserved populations, including people with disabilities (PWD) who remain largely overlooked in this field.

The ADA (Americans with Disabilities Act, 1990) defines a person with a disability as “one who has a physical or mental impairment that substantially limits one or more major life activities, who has a history or record of such an impairment, or who is perceived by others as having such an impairment.” Beyond clinical diagnoses, disability is increasingly being recognized as a social and functional construct, or a dynamic interaction between the individual and societal or environmental barriers (Shakespeare, 2013; World Health Organization, 2002, 2025). This perspective underscores the relevance of individuals’ self-identification when studying disability-related disparities in health outcomes. While findings suggest that nearly one-third of the population has a disability (CDC, 2024), there is a striking lack of empirical research regarding the mental health and substance use needs of this population (DeCormier Plosky et al., 2022). Although many public health issues, including SUDs, disproportionately affect PWD, research and intervention frameworks rarely acknowledge or accommodate the population (Krahn, Walker, & Correa-De-Araujo, 2015). In the field of substance use research, for example, the National Survey on Drug Use and Health (NSDUH), a primary data source guiding national substance use policy, excludes unhoused and institutionalized individuals—groups with elevated disability rates, thus limiting its capacity to capture disability-related disparities (de Sousa et al., 2023; World Health Organization, 2022). As a result, substance use patterns and treatment barriers faced by PWD remain poorly understood.

These literature gaps and survey exclusions are alarming given that PWD are significantly more likely to experience psychological distress (Cree, 2020) and to develop SUDs compared to the general population (Shearer, Howell, Bart, & Winkelman, 2020). In a study of Medicaid enrollees, PWD were 14–16 % more likely to have a SUD compared to those without disabilities (Roux, Tao, Marcus, Lushin, & Shea, 2022). Although Medicare eligibility for individuals under age 65 is typically based on disability status, this group accounted for a striking 80.8 % of opioid overdose deaths among all Medicare enrollees, highlighting an urgent concern at the intersection of disability, age, and overdose risk (Kuo, Raji, & Goodwin, 2019). However, PWD utilize substance use rehabilitation services at approximately half the usage rates of individuals without disabilities (Hinson-Enslin, Nahhas, & McClintock, 2022).

Prior evidence has documented associations between disability and negative health-related consequences, such as depression, anxiety, chronic pain, and lifetime trauma. In turn, these factors are associated with higher rates of substance use and SUDs (Chapman & Wu, 2012; Reif et al., 2022). Moreover, PWD experience stressors related to stigmatization and systemic barriers (e.g., social exclusion, inadequate support services) further complicating their utilization, access, and quality of SUD treatment (Reif et al., 2022). Despite these critical needs, PWD are less likely to receive mental health and/or substance use assessments by providers (Iezzoni & O’Day, 2006), which could be due to mistaken assumptions that PWD are less likely to use substances or are unlikely to benefit from recovery interventions (Ledingham, Adams, Heaphy, Duarte, & Reif, 2022). Altogether, these findings indicate that structural stigma and inadequate provider training often limit PWD access to equitable SUD care.

Further, there is a critical need for a deeper understanding of substance use and recovery outcomes in PWD to support clinicians and addictions researchers in identifying opportunities for proactive intervention in substance use. While existing literature documents prevalence of substance use in PWD, most studies have utilized qualitative methods, administrative data, or focused on specific disability subgroups, thus leaving a gap in comparative, empirical analyses of recovery experiences across disability status in treatment-seeking community populations. The present study seeks to address this gap in exploring the following research questions: (1) how does substance use recovery trajectory vary across self-reported disability status; (2) among people who use substances, are there different rates in mental health comorbidities across disability status; and (3) what barriers to substance use treatment services disproportionately affect PWD? The present study is the first to our knowledge to investigate how recovery outcomes, mental health comorbidities, and barriers to care differ across disability status using structured survey data among people who use substances.

## 2. Methods

### 2.1. Sample and recruitment procedures

This study utilized baseline data from a population sample enrolled in a digital intervention (uMAT-R) aimed at supporting the recovery of adults with substance use disorder (Cavazos-Rehg et al., 2020). Two questions regarding disability status were added to the initial baseline survey in March 2023. Between March 2023 and December 2024, 350 participants had responded to the item. We recruited participants from various addiction treatment facilities within Missouri, as well as recovery homes, justice settings, emergency rooms, and also through snowball sampling (see Appendix A). Eligibility criteria included: 1) a history of opioid, stimulant, or hallucinogen misuse; 2) age 18 or older; 3) U.S. residency; 4) English-speaking; and 5) smartphone ownership. After consenting, participants completed a baseline survey and were granted access to the uMAT-R application. All data points reviewed in the present study are those from the baseline survey. All baseline data were collected online via self-report surveys on the Research Electronic Data Capture system (REDCap). The WashU Medicine Institutional Review Board approved the present study (#201910161).

### 2.2. Measures

#### 2.2.1. Disability status

In the baseline survey, participants self-reported, “Do you identify as having a disability? Disability is defined by the ADA as a physical or mental impairment that substantially limits one or more life activities.” Response options included “no,” “yes,” and “prefer not to answer.” We used self-reported disability status given the lack of a standardized disability identification method (Hall, Kurth, Ipsen, Myers, & Goddard, 2022), its alignment with the ADA’s inclusion of perceived limitations (Americans with Disabilities Act, 1990), and the reliable correspondence of self-report with administrative determinations (Benítez-Silva, Buchinsky, Man Chan, Cheidvasser, & Rust, 2004). The use of self-report to assess disability status also mirrors prior methodologies in studies investigating SUD in PWD (Ledingham et al., 2022; Reif et al., 2022), which similarly prioritize social and functional constructs of disability.

Participants could also disclose their specific type of disability in response to the prompt: “Please specify the kind of disability or disabilities you have. Examples: physical disability, learning disability, invisible illness.” Based on the lack of standardization in the open-ended responses given, we were unable to systematically categorize subtypes of disability beyond preliminary categories.^1^ We further explore the implications of this methodology in the limitations section.

#### 2.2.2. Sociodemographic characteristics

The baseline survey queries age, gender (female vs. male vs. nonbinary), race and ethnicity (white vs. people of color), educational attainment (below high school vs. high school equivalent or above), employment status (unemployed vs. any employment), living arrangement (unhoused/unstably housed vs. stably housed), insurance coverage (uncovered vs. covered), and incarceration history (yes vs. no).

#### 2.2.3. Recent and past-year substance use history

We assessed past 30-day use of cannabis, alcohol, opioids, stimulants, and other illicit drugs independently using a single-item question with response options: “not at all,” “a few times,” “a few times each week,” and “every day.” For analysis, we dichotomized these responses as “no” (“not at all”) versus “yes” (“a few times,” “a few times each week,” “every day”) for each substance.

We took questions assessing symptoms of SUD from the NSDUH (Daniel, Richesson, Magas, Brown, & Hoenig, 2022), which align with DSM-5 criteria (American Psychiatric Association, 2022). We additionally created a new measure (a count of all applicable criteria) to measure the severity of SUD symptoms endorsed by participants over the past 12 months. Consistent with existing literature, we defined the severity as follows: none (0–1 symptoms); mild (2–3); moderate (4–5); and severe (≥6) (Center for Behavioral Health Statistics and Quality, 2021). We calculated SUD severity scores for opioids, stimulants, and hallucinogens, consistent with the study’s eligibility criteria. Although we assessed past 30-day alcohol and cannabis use, DSM-5-based severity scoring was not assessed for alcohol or cannabis use disorders in the baseline survey.

#### 2.2.4. Mental health comorbidities

##### Depressive symptoms.

The Patient Health Questionnaire-9 (PHQ-9) (Kroenke, Spitzer, Williams, & Löwe, 2009) measured depression symptoms during the preceding two weeks. We differentiated between no to mild symptoms (scores 0 to 9) and moderate to severe symptoms (scores 10 to 27). *Anxiety symptoms.* The Generalized Anxiety Disorder-7 (GAD-7) (Spitzer, Kroenke, Williams, & Löwe, 2006) measured anxiety symptoms in the same format as the PHQ-9. Scores ranging from 0 to 9 were rated as mild symptoms, while scores from 10 to 21 were rated as moderate-severe. *Lifetime suicide attempt.* Participants responded to a “yes/no” question: “Have you ever, at any point in your life, tried to end your own life or made an attempt at suicide?”

#### 2.2.5. Clinical and recovery experiences

Participants were asked “How many times have you overdosed by drug?” Participants could answer “Never/1–3 times/4–5 times/6–7 times/8–10 times/11 or more times.” For analysis, we dichotomized responses as “0 (never)” to “1 or more times.” We assessed history of recovery attempts by asking, “How many times have you been in recovery prior to starting here at your current treatment facility?” Options included a range from “1^st^ time in recovery” to “5^th^ time in recovery,” as well as “I’ve been in recovery more than 5 times.” For analysis, we dichotomized the responses as 3 or fewer recovery attempts versus 4 or more attempts. Participants were asked, “Did you miss any appointments or group sessions in the past month?” with response options of “yes” or “no.”

#### 2.2.6. Barriers to care & unmet needs

**Fig. 1.**
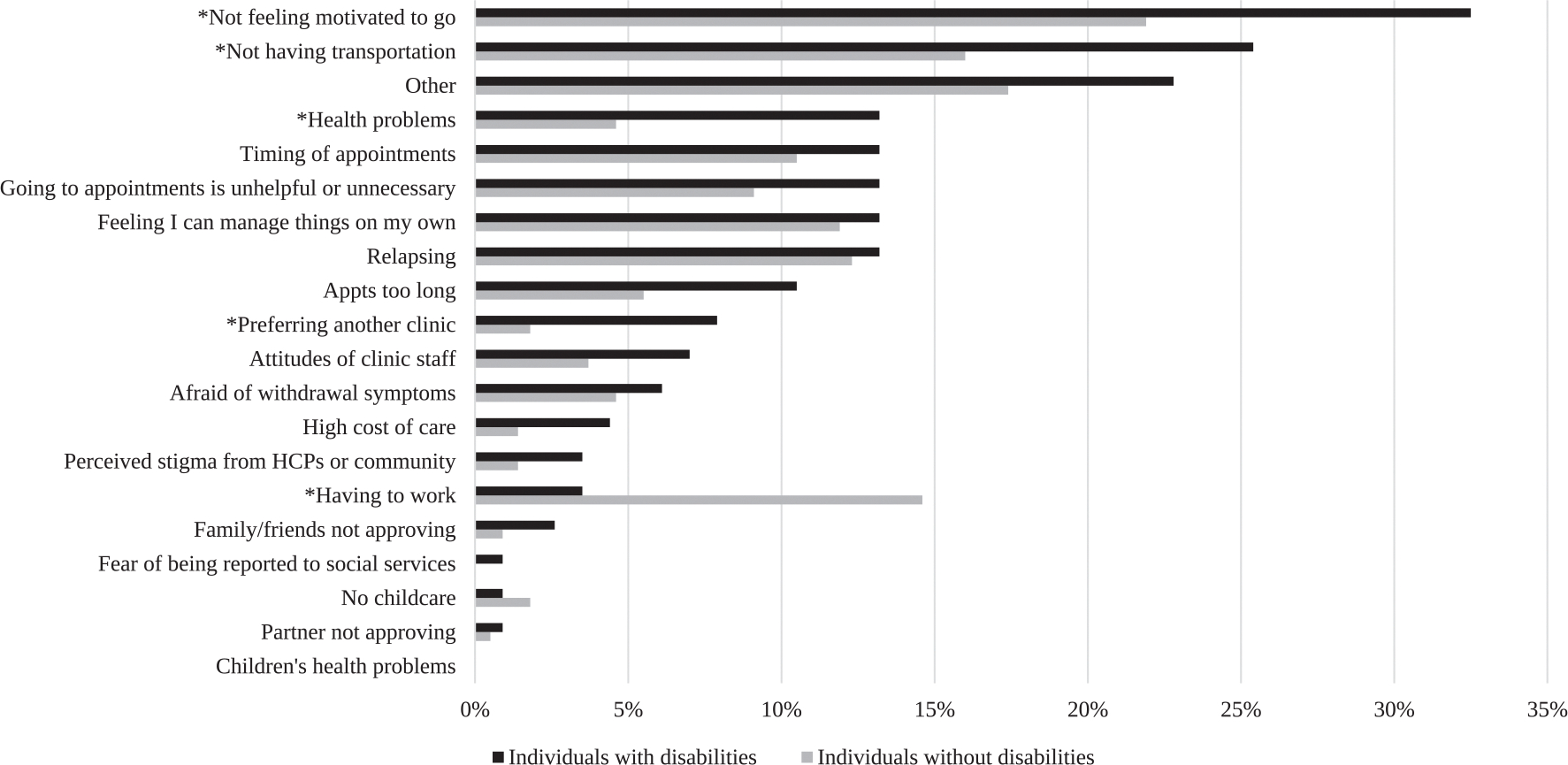
Barriers to Substance Use Treatment Endorsed by Participants With and Without Self-Reported Disabilities. P-values were calculated using Pearsons CHISQ tests. Fisher Exact were applied when the cell size was less than 5. *[Barrier Label] indicates statistically significant difference. The ‘Other’ category reflects open-text responses provided by participants whose reasons for missing treatment were not captured by predefined options (e.g., “social anxiety”, “tired,” “don’t want to get urinalysis”).”

**Fig. 2.**
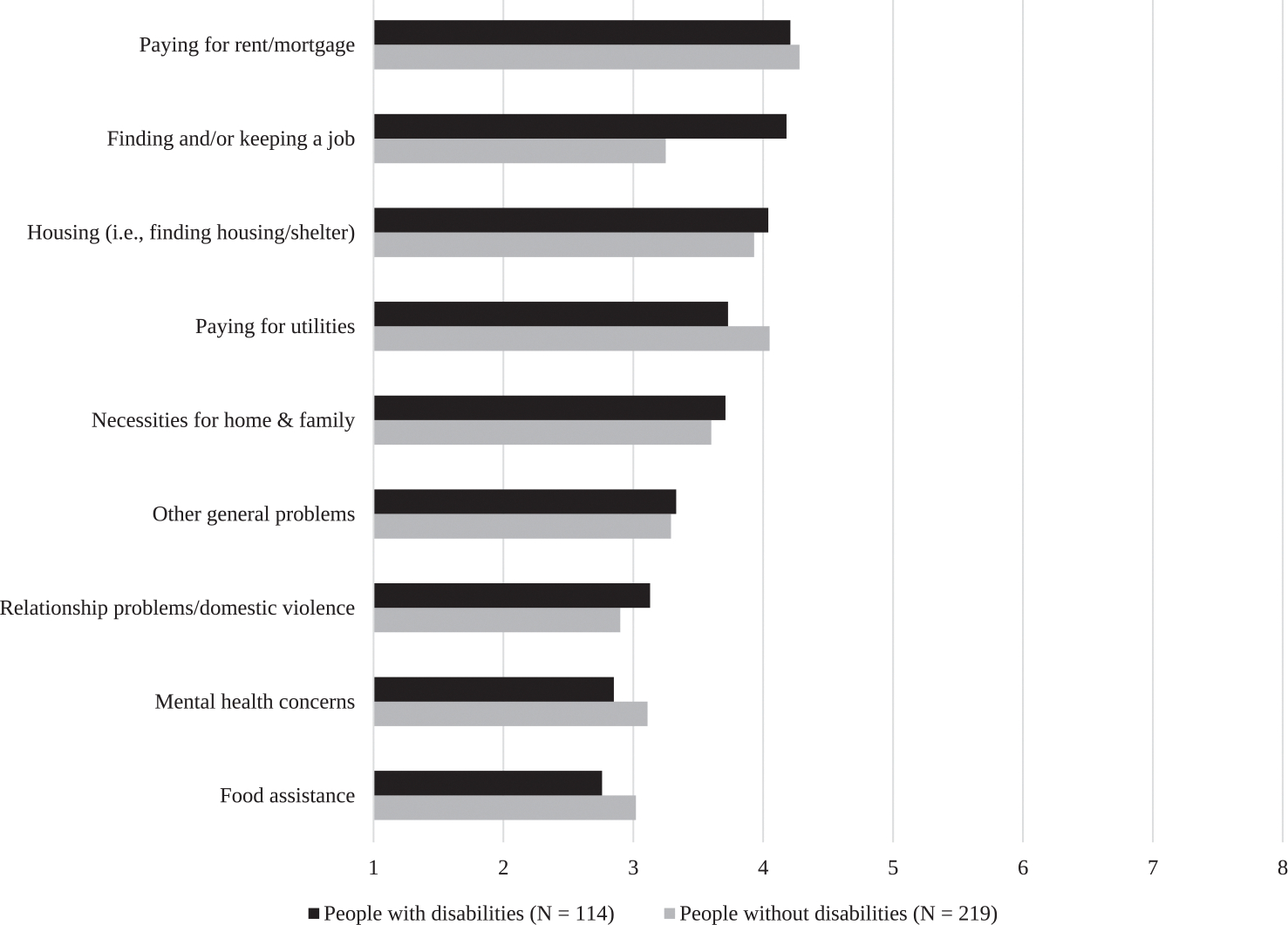
Unmet Needs Endorsed by Participants with and Without Self-Reported Disabilities. Rating Scale: 1 (Very Confident) - 8 (Not Confident At All).

Participants selected all that applied from a list of 20 reasons for missing appointments (Fig. 1), representing barriers to substance use treatment (Cachelin & Striegel-Moore, 2006; Szlyk, Dell, Li, Winograd, & Cavazos-Rehg, 2025). We considered each barrier individually in analysis and compared the total number of barriers marked by each participant as well. We adapted an additional measure of Unmet Basic Needs from the Wellbeing and Basic Needs Survey (Zuckerman, 2020). Specifically, a 9-item measure evaluated participants’ confidence in their ability to access essentials such as food, shelter, and safety resources (Fig. 2). Participants responded on a scale ranging from “very confident” (1) to “not confident at all.” (8). Total scores on the measure ranged from 9 to 72, with higher scores indicating a greater degree of unmet basic needs.

### 2.3. Statistical data analysis

Descriptive statistics were reported for the entire sample and based on self-reported disability status. Bivariate analyses were conducted to examine differences between individuals with and without disabilities. Independent *t*-tests were conducted for continuous variables (e.g., barriers to care, unmet needs) while Pearson’s Chi-squared tests were performed for categorical variables (e.g., recent substance use). When the cell size was less than 5, Fisher’s exact test was performed.

To quantify the association between outcomes of interest and disability status, multivariable regression models were conducted, controlling for sociodemographic covariates. Specifically, logistic regression was performed for categorical outcomes (e.g., recent substance use), and linear regression was performed for continuous outcomes (e.g., barriers to care, unmet needs). We reported the adjusted odds ratios (aORs) along with 95 % confidence intervals (CIs) for logistic models or coefficients (βs) and standard errors (SEs) for linear models.

All analyses were performed using SAS Version 9.4. All statistical analyses were two-sided and *p* values <0.05 were considered statistically significant.

## 3. Results

### 3.1. Disability status

350 individuals completed the baseline survey during the defined study period. 333 participants answered the question regarding disability status with either a “yes” or a “no,” and 17 participants declined to answer. The 17 participants who declined to state their disability status were excluded from the analysis. Among the participants, 219 individuals did not have disabilities (65.8 %), while 114 individuals reported having a disability (34.2 %).

### 3.2. Sociodemographic characteristics (Table 1)

The mean age of participants was 41.1 years (SD = 10.6), with 192 females (58.0 %) and 105 people of color (31.8 %). Participants with disabilities were older compared to those without disabilities (M_age_ = 45.3 (SD = 11.3) vs. 39.0 (9.7); *p* < 0.001). A greater proportion of PWD were people of color (44.6 % vs. 25.2 %; *p* < 0.001), unemployed (82.7 % vs. 55.5 %; *p* < 0.001), and covered by insurance, including government-funded health insurance (96.5 % vs. 84.5 %; *p* = 0.001). There was no significant difference in disability prevalence between recruitment sites (*p* = 0.10; see Appendix A).

### 3.3. Substance use history (Table 2)

When examining drug use during the 30-days preceding the survey, we found statistically significant differences across disability status. Specifically, a higher proportion of respondents with disabilities reported use of opioids (28.9 % vs. 17.3 %; *p* = 0.01), stimulants (27.4 % vs. 9.6 %; *p* < 0.001), non-opioid illicit drugs (29.0 % vs. 12.8 %; *p* < 0.001), cannabis (26.3 % vs. 15.1 %; *p* = 0.01), and alcohol (27.2 % vs. 13.8 %; *p* = 0.003) during the past 30 days. No difference was observed in the severity of SUD symptoms across opioids and stimulants, though more PWD met criteria for severe hallucinogen use disorder (43.1 % vs. 14.7 %; *p* = 0.001).

In adjusted analyses, multivariable logistic regression results suggested that PWD had 2-times or higher odds of using stimulants, non-opioid illicit drugs, cannabis, and alcohol, compared to those without disabilities (aOR (95 % CI): 2.30 (1.12, 4.75); 2.63 (1.34, 5.16); 2.33 (1.22, 4.43); and 2.22 (1.14, 4.32), respectively). Additionally, compared to those without disabilities, PWD were more likely to meet criteria for severe hallucinogen use disorder (aOR (95 % CI): 4.84 (1.82, 12.92)).

### 3.4. Mental health comorbidities (Table 3)

About a third of the sample endorsed moderate to severe depression (*n* = 121, 37.5 %) and anxiety (*n* = 101, 31.3 %), respectively. Nearly half of participants had attempted suicide during their lifetime (*n* = 154, 46.7 %). PWD were more likely to report moderate to severe depression compared to participants without disabilities (55.0 % vs. 28.5 %; *p* < 0.001), as well as anxiety (42.7 % vs. 25.3 %; *p* = 0.001) and a history of suicide attempts (58.4 % vs. 40.5 %; *p* = 0.002). In the adjusted model, PWD had significantly higher odds of reporting moderate to severe depression (aOR (95 % CI): 3.38 (1.91, 5.99)), anxiety (2.39 (1.32, 4.32)), and a history of suicide attempt (2.56 (1.48, 4.43)) compared to those without disabilities.

### 3.5. Clinical and recovery experiences (Table 4)

Nearly half of all participants reported having experienced an overdose in their lifetime (*n* = 158, 47.4 %) and more than half reported having undergone 4 or more recovery attempts (*n* = 187, 56.2 %). Results from logistic regression suggested that PWD were at 1.60 higher odds of having experienced an overdose in their lifetime compared to those without disabilities (aOR (95 % CI): 2.60 (1.49, 4.54)), when controlling for sociodemographic covariates. PWD reported significantly more recovery attempts (e.g., 4 times or more) compared to those without disabilities (68.4 % vs. 49.8 %; p = 0.001), which was affirmed in the adjusted model (aOR (95 % CI): 2.69 (1.55, 4.68)). About one-third of all participants missed an appointment or group session in the past 30 days (*n* = 100, 30.2 %). PWD were more likely to miss treatment appointments compared to those without disabilities (37.7 % vs. 26.3 %; *p* = 0.03), though there was no significant association after adjusting for sociodemographic covariates.

### 3.6. Barriers to care (Fig. 1) & unmet needs (Fig. 2)

PWD identified more barriers to care than those without disabilities (Mean (SD): 1.9 (2.3) vs 1.3 (1.6); *p* = 0.003); in the adjusted model, the difference in number of barriers to care remained significant (β (SE): 0.55 (0.24)). PWD more commonly endorsed barriers to care including lack of motivation, lack of transportation, comorbid health problems, and preferring another clinic. In contrast, those without disabilities more frequently endorsed ‘having to work’ as a barrier to care, consistent with the significant difference in employment rates by disability status in our participants. No significant difference was found in meeting basic needs across disability status.

## 4. Discussion

This is the first study to our knowledge to highlight differences in substance use recovery outcomes, mental health comorbidities, and barriers to recovery across self-reported disability status among a community-based sample of people who use substances. Strikingly, PWD who use substances reported more recovery attempts, missed more appointments, exhibited higher rates of depressive and anxiety symptoms, had more lifetime suicide attempts, and faced greater barriers to care than patients without disabilities, even after controlling for sociodemographic covariates. Our findings highlight the significant impact that disability status has on substance use recovery trajectory, underscore the importance of comprehensive screening for substance use and associated risk factors in PWD, and emphasize the need for more research in this area to better understand how to lessen these inequities.

We observed significant differences in recent substance use across all of the investigated substances, in which PWD were more likely to have used each substance in the past 30 days. Our findings thus align with previous results regarding cannabis (Glazier & Kling, 2013; Yang et al., 2023); alcohol (Aram, Slopen, Arria, Liu, & Dallal, 2023); stimulants (Casseus et al., 2021; Deutsch & Burket, 2021); opioids (Glazier & Kling, 2013; Reif et al., 2022), and the general group of “all other substances” (Czeisler, 2021; Schulz, Gimm, West, Kock, & Villanti, 2024). Further, in the adjusted model, we found disability to be a distinct vulnerability marker for overdose risk. This pattern aligns with national findings showing elevated overdose mortality risk among adults with disabilities (Aram et al., 2024; Kuo et al., 2019) and suggests that structural disadvantage may obscure the true burden of overdose risk in PWD unless appropriately accounted for.

Although we did not find significant differences in overall SUD prevalence rates between PWD and non-disabled peers, this discrepancy with prior studies (Aram et al., 2024; Kuo et al., 2019; Mills, 2023) may reflect our sample’s composition—participants with substance misuse history who were engaged in or seeking treatment—which may have attenuated diagnostic prevalence differences. These findings reinforce the need for clinicians to assess for substance use in PWD, as their baseline risk is higher than for patients without disabilities.

Further, our findings revealed that PWD were more likely to experience depressive and anxiety symptoms in recent weeks, aligning with existing evidence (Cree, 2020; Tough, Siegrist, & Fekete, 2017). When such disorders co-occur with substance use, symptoms can be protracted, severe, and resistant to treatment, begetting worse health outcomes (DeMarce, Lash, Stephens, Grambow, & Burden, 2008; Krawczyk et al., 2017; Santucci, 2012). Further, our sample had a significantly elevated history of suicide attempts, especially in PWD. It has been well-documented that suicide rates are elevated in people who use substances (up to 52.1 %) (Leza, Haro, López-Goñi, & Fernández-Montalvo, 2024) and in PWD (Marlow, Xie, Tanner, Jo, & Kirby, 2021). The combination of substance use and disability may have compounding effects on vulnerability to suicidal behavior. Given the strong association between substance use, mood/anxiety disorders (Lai, Cleary, Sitharthan, & Hunt, 2015), and suicidal behaviors, and the increased prevalence of all these in PWD, clinicians should routinely assess for both mental health concerns and substance use when treating PWD.

Regarding SUD treatment history, PWD in our study population reported more recovery attempts and were more likely to have missed a treatment appointment in the past 30 days. However, the association between disability status and missed appointments did not remain statistically significant after adjusting for sociodemographic covariates—particularly employment, which differed significantly across disability status. These findings remain clinically meaningful and align with prior research documenting higher rates of treatment disengagement among PWD (Kroll, Jones, Kehn, & Neri, 2006; Thomas et al., 2023). While the cross-sectional data cannot determine direction of causation between missed appointments and number of recovery attempts, it stands to reason that disruptions in treatment engagement—whether due to comorbid health conditions, structural stigma, or access barriers—may contribute to repeated cycles of relapse and recovery (Milward, Lynskey, & Strang, 2014; Molfenter, 2013). Supporting appointment adherence may therefore be an important target for improving recovery outcomes in this population.

Furthermore, among our study population, PWD faced more barriers to care than those without disabilities, including lack of motivation to attend appointments, preferring to be in another clinic, not having transportation, and comorbid health problems. The preference for another clinic and low motivation to attend appointments could indicate perceived or actual stigmatization by staff, suboptimal cultural competence in treatment programs, or concerns about accessibility and adequacy of services (Caris & Beckers, 2022; Gibson & O’Connor, 2010; Iezzoni et al., 2021). It has been well-established that PWD face increased prevalence of comorbid chronic illnesses (National Council on Disability, 2022), as well as transportation barriers limiting their access to community resources (Bezyak et al., 2020). Addressing these barriers is essential for improving SUD treatment engagement and outcomes in PWD. Further research can provide insights into these barriers and inform targeted interventions to enhance the accessibility and quality of substance use treatment for PWD.

## 5. Limitations

In the present study, our qualification of participants’ psychiatric symptoms and substance use relied on self-report. Self-report bias may be especially relevant in PWD, who may have heightened sensitivity to social perceptions and stigma. Further, PWD may have been less likely to find the survey methods or language accessible. Also of note, our cross-sectional data from the baseline survey limits the potential to understand causal relationship between disability onset and substance use. Selection bias can affect recruitment, as researchers recruited participants from substance use treatment settings, suggesting that respondents were more likely to be motivated to seek recovery support for substance use. Mobile app-based research can dissuade participation of those who are less technologically savvy (commonly those who are older, in rural areas, impoverished, or otherwise disadvantaged) (Dorsey, McConnell, Shaw, Trister, & Friend, 2017). Additionally, we collapsed multiple racial and ethnic subgroups into a single “people of color” category due to small sample sizes, which may have obscured important differences between these groups.

While the findings suggest multiple significant associations between disability status and substance use patterns, there were limitations due to the variability in how participants conceptualized disability. Existing instruments do not define disability status in a uniform manner; as such, researchers have used various approaches to identify people with disabilities within their studies, including administrative and insurance records, or self-report through surveys and assessment tools (Ryding, Scales, Brittingham, & Holz, 2022). Such self-report methodology has been established as a reliable measure of disability status (Benítez-Silva et al., 2004), which is why prior studies investigating the intersection of substance use and disability status have also relied on self-report (Ledingham et al., 2022; Reif et al., 2022).

Further, while our use of a binary self-report disability measure was practical for initial analysis, it did not allow for analysis of relationships between disability types and substance use or recovery trajectories. We did collect qualitative descriptions of participants’ disabilities, though the responses were highly heterogeneous and lacked specificity,^1^ thus precluding more granular analysis. Future research should include a more defined classification scheme of disability subtypes. Other disability health researchers have affirmed the need for a better defined classification scheme of disability subtypes in the literature and posit that future efforts to standardize classifications of disability across the field of scientific investigation (Landes et al., 2024) may support our understandings of how specific types of disabilities (e.g., sensory, physical, cognitive) are differentially associated with mental health concerns and substance use patterns.

## 6. Conclusion

In the present study, people with self-reported disabilities were more likely to report recent substance use of all types, mental health comorbidities, barriers to treatment, and more protracted recovery processes compared to those without disabilities. These disparities across disability status underscore the need for proactive screening and intervention strategies to mitigate these risks. By implementing targeted, accessible, and culturally competent care models, we can begin to address disparities in substance use treatment outcomes for PWD. Future research should explore how mental health conditions, substance use patterns, and recovery outcomes vary across disability subtypes, thus guiding the development of tailored, person-centered treatment approaches. By addressing barriers to care and ensuring equitable access to recovery services, we can begin to bridge the healthcare gap for PWD, ultimately improving both substance use outcomes and their overall well-being.

## Figures and Tables

**Table 1:** Sociodemographic characteristics between participants without and with disabilities.

	Overall (*N* = 333)	Individuals without disabilities (*N* = 219, 65.8 %)	Individuals with disabilities (*N* = 114, 34.2 %)	*p*
	N (%)			

Age, Mean (SD)	41.1 (10.6)	39.0 (9.7)	45.3 (11.3)	**<0.001**
Gender				
Male	133 (40.2)	85 (39.0)	48 (42.5)	0.17
Female	192 (58.0)	131 (60.1)	61 (54.0)	
Others^a^	6 (1.8)	2 (0.9)	4 (3.5)	
Race & Ethnicity				
People of color^b^	105 (31.8)	55 (25.2)	50 (44.6)	**<0.001**
White Education	225 (68.2)	163 (74.8)	62 (55.4)	
Below high school	64 (19.5)	42 (19.3)	22 (19.8)	0.90
High school equivalent & above	265 (80.6)	176 (80.7)	89 (80.2)	
Employment status				
Unemployed	212 (64.6)	121 (55.5)	91 (82.7)	**<0.001**
Employed (including part-time)	116 (35.4)	97 (45.5)	19 (17.3)	
Living arrangement				
Homeless or unstable housing^c^	193 (58.0)	127 (58.0)	66 (57.9)	0.99
Living in home/apartment	140 (42.0)	92 (42.0)	48 (42.1)	
Insurance				
Uncovered	38 (11.4)	34 (15.5)	4 (3.5)	**0.001**
Covered	295 (88.6)	185 (84.5)	110 (96.5)	
Incarceration				
No	125 (37.8)	80 (36.5)	45 (40.2)	0.52
Yes	206 (62.2)	139 (63.5)	67 (59.8)	
If yes, drug-related crime				
No	53 (25.9)	29 (21.0)	24 (35.8)	0.02
Yes	152 (74.1)	109 (79.0)	43 (64.2)	

Numbers may not sum to 333 (100 %) due to missing data.

P-values were calculated using Pearsons CHISQ-test.

**Bold** indicates statistically significant.

a In this category, 3 individuals (50.0 %) identified as non-binary, and 3 individuals (50.0 %) responded “Other.”

b In this category, 82 individuals (78.1 %) identified as African American/Black, 10 (9.5 %) as Hispanic, 3 (2.9 %) as American Indians or Alaska Native, 2 (1.9 %) as Asians, 8 as others.

c Unstable housing indicates the individual currently lives in shelter, on the street, treatment facility, medical center, or someone else’s apartment/house.

**Table 2:** Substance use history in participants without and with disabilities.

	Overall (N = 333)	Individuals without disabilities (Ref) (N = 219, 65.8 %)	Individuals with disabilities (N = 114, 34.2 %)	*p*	aOR (95 % CI)^a^
		
	N (%)				

Past 30-day opioid use					
No	262 (78.7)	181 (82.7)	81 (71.1)	**0.01**	Ref
Yes	71 (21.3)	38 (17.3)	33 (28.9)		**1.32 (0.70, 2.48)**
Past 30-day stimulant use					
No	279 (84.3)	197 (90.4)	82 (72.6)	**<0.001**	Ref
Yes	52 (15.7)	21 (9.6)	31 (27.4)		**2.30 (1.12, 4.75)**
Past 30-day other drug use					
No	272 (81.7)	191 (87.2)	81 (71.0)	**<0.001**	Ref
Yes	61 (18.3)	28 (12.8)	33 (29.0)		**2.63 (1.34, 5.16)**
Past 30-day cannabis use					
No	270 (81.0)	186 (84.9)	84 (73.7)	**0.01**	Ref
Yes	63 (19.0)	33 (15.1)	30 (26.3)		**2.33 (1.22, 4.43)**
Past 30-day alcohol use					
No	271 (81.6)	188 (86.2)	83 (72.8)	**0.003**	Ref
Yes	61 (18.4)	30 (13.8)	31 (27.2)		**2.22 (1.14, 4.32)**
Past-year DSM-5 Opioid use disorder					
None (0 or 1 symptom)	98 (43.4)	75 (48.4)	23 (32.4)	0.12	Ref
Mild (2 or 3 symptoms)	10 (4.4)	5 (3.2)	5 (7.0)		
Moderate (4 or 5 symptoms)	6 (2.7)	4 (2.6)	2 (2.8)		
Severe (6 or more symptoms)	112 (49.6)	71 (45.8)	41 (57.8)		1.54 (0.79, 2.97)
Past-year DSM-5 Stimulant use disorder					
None (0 or 1 symptom)	100 (35.8)	76 (40.9)	24 (25.8)	0.07	Ref
Mild (2 or 3 symptoms)	19 (6.8)	11 (5.9)	8 (8.6)		
Moderate (4 or 5 symptoms)	12 (4.3)	6 (3.2)	6 (6.5)		
Severe (6 or more symptoms)	148 (53.1)	93 (50.0)	55 (59.1)		1.21 (0.68, 2.16)
Past-year DSM-5 Hallucinogen use disorder					
None (0 or 1 symptom)	117 (73.1)	88 (80.7)	29 (56.9)	**0.001**	Ref
Mild (2 or 3 symptoms)	3 (1.9)	3 (2.8)	0		
Moderate (4 or 5 symptoms)	2 (1.3)	2 (1.8)	0		
Severe (6 or more symptoms)	38 (23.8)	16 (14.7)	22 (43.1)		**4.84 (1.82, 12.92)**

Ref, Reference; aOR, adjusted Odds Ratio; CI, Confidence Interval.

P-values were calculated using Pearsons CHISQ-test.

**Bold** indicates statistically significant.

a The estimates were obtained using multivariable logistic regression, controlling for socio-demographics (e.g., age, gender, race & ethnicity, educational attainment, employment status, living arrangement, and insurance coverage).

**Table 3:** Mental health comorbidities between participants without and with disabilities.

	Overall (N = 333)	Individuals without disabilities (Rei) (N = 219, 65.8 %)	Individuals with disabilities (N = 114, 34.2 %)	*p*	aOR (95 % CI)^a^
		
	N (%)				

Depression^b^					
None/mild	202 (62.5)	153 (71.5)	49 (45.0)	**<0.001**	Ref
Moderate/severe	121 (37.5)	61 (28.5)	60 (55.0)		**3.38 (1.91, 5.99)**
Anxiety^b^					
None/mild	222 (68.7)	159 (74.7)	63 (57.3)	**0.001**	Ref
Moderate/severe	101 (31.3)	54 (25.3)	47 (42.7)		**2.39 (1.32, 4.32)**
Lifetime Suicide attempts					
No	176 (53.3)	129 (59.5)	47 (41.6)	**0.002**	Ref
Yes	154 (46.7)	88 (40.5)	66 (58.4)		**2.56 (1.48, 4.43)**

Ref, Reference; aOR, adjusted Odds Ratio; CI, Confidence Interval.

*P*-values were calculated using Pearsons CHISQ-test.

**Bold** indicates statistically significant.

a The estimates were obtained using multivariable logistic regression, controlling for socio-demographics (e.g., age, gender, race & ethnicity, educational attainment, employment status, living arrangement, and insurance coverage).

b Depression and Anxiety were assessed by using PHQ-9 and GAD-7, respectively. A cut-off of 9 was applied to dichotomized none/mild vs. moderate/se-vere depression or anxiety.

**Table 4:** Treatment history and factors influencing treatment engagement by disability status.

	Overall (*N* = 333)	Individuals without disabilities (Ref) (N = 219, 65.8 %)	Individuals with disabilities (N = 114, 34.2 %)	*p*	aOR (95 % CI)^a^/β (SE)^b^
		
	N (%)				

Lifetime overdose					
No	175 (52.6)	123 (56.2)	52 (45.6)	0.07*	Ref
Yes	158 (47.4)	96 (43.8)	62 (54.4)		**2.60 (1.49, 4.54)**
Recovery attempt^c^					
1–3 times	146 (43.8)	110 (50.2)	36 (31.6)	**0.001** *	Ref
4 times or more	187 (56.2)	109 (49.8)	78 (68.4)		**2.69 (1.55, 4.68)**
Missed an appointment or group session during past 30-day					
No	231 (69.8)	160 (73.7)	71 (62.3)	**0.03** *	Ref
Yes	100 (30.2)	57 (26.3)	43 (37.7)		1.64 (0.93, 2.88)
Number of barriers to treatment endorsed^d^, Mean (SD)	1.5 (1.9)	1.3 (1.6)	1.9 (2.3)	**0.003** **	**0.55 (0.24)**
Unmet basic needs, Mean (SD)	3.5 (2.0)	3.5 (2.0)	3.6 (2.1)	0.81**	0.05 (0.26)^d^

Ref, Reference; aOR, adjusted Odds Ratio; CI, Confidence Interval; SE, Standard Error; SD, Standard Deviation.

**Bold** indicates statistically significant.

a The estimates (i.e., aOR (95 % CI)) were obtained using multivariable logistic regression, controlling for socio-demographics (e.g., age, gender, race & ethnicity, educational attainment, employment status, living arrangement, and insurance coverage).

b The estimates (i.e., β (SE)) were obtained using multivariable linear regression, controlling for socio-demographics (e.g., age, gender, race & ethnicity, educational attainment, employment status, living arrangement, and insurance coverage).

c The provided options for the item assessing recovery attempts range from 1 to 6 or more. The Row Mean Score Test was also performed to compare mean scores by disability status. The *p*-value obtained from the test was 0.01, indicating statistically significant.

d One barrier, “Having to work,” was excluded from the analysis because of the high proportion of unemployment among individuals with disabilities. Therefore, the total number of barriers to treatment included in the regression model was 19.

* P-values were calculated using Pearsons CHISQ-test.

** P-values were calculated using Independent t-test.

## Footnotes

1 92 participants self-identified a total of 147 disabling conditions and 22 participants who had said “yes” to the question of having a disability did not provide further context. 89 of the disabilities listed were specific diagnoses, while the remaining 58 were listed as a subtype (e.g., “physical” (21); “invisible illness” (11); “mental health”/“mental illness” (11); “mental disability” (4); “mental” (11)). Concerns of ambiguity were related to the multiple phrasings of “mental” conditions (possibly representing psychiatric or cognitive disabilities), and for many specific diagnoses, such as traumatic brain injury (reported by 3 participants), which could fit into physical and/or cognitive disability subtypes.

## Appendix A. Recruitment location by disability status

**Table:**

	Overall (N = 333)	Individuals without disabilities (N = 219, 65.8 %)	Individuals with disabilities (*N* = 114, 34.2 %)	*p*
		
	N (%)			

Recruited from				
Treatment facility/Recovery home	237 (71.1 %)	157 (71.7)	80 (70.2)	0.10
Emergency department	43 (13.0)	22 (10.0)	21 (18.4)	
Justice setting	5 (2.0)	4 (1.8)	1 (0.9)	
Self-referral	48 (14.4)	36 (16.4 %)	12 (10.5)	
